# High-frequency and high-quality silicon carbide optomechanical microresonators

**DOI:** 10.1038/srep17005

**Published:** 2015-11-20

**Authors:** Xiyuan Lu, Jonathan Y. Lee, Qiang Lin

**Affiliations:** 1 Department of Physics and Astronomy, University of Rochester, Rochester, NY 14627, USA; 2 Department of Electrical and Computer Engineering, University of Rochester, Rochester, NY 14627, USA; 3 Institute of Optics, University of Rochester, Rochester, NY 14627, USA

## Abstract

Silicon carbide (SiC) exhibits excellent material properties attractive for broad applications. We demonstrate the first SiC optomechanical microresonators that integrate high mechanical frequency, high mechanical quality, and high optical quality into a single device. The radial-breathing mechanical mode has a mechanical frequency up to 1.69 GHz with a mechanical Q around 5500 in atmosphere, which corresponds to a f_m_ · Q_m_ product as high as 9.47 × 10^12^ Hz. The strong optomechanical coupling allows us to efficiently excite and probe the coherent mechanical oscillation by optical waves. The demonstrated devices, in combination with the superior thermal property, chemical inertness, and defect characteristics of SiC, show great potential for applications in metrology, sensing, and quantum photonics, particularly in harsh environments that are challenging for other device platforms.

Optomechanical resonators couple optical cavities and mechanical resonators mutually through optomechanical interactions mediated by the radiation-pressure forces. With the exceptional capability of probing and controlling mesoscopic mechanical motion down to single quantum level, micro/nano-optomechanical resonators have been intensively investigated in recent years, showing great promise for broad applications in sensing, information processing, time/frequency metrology, and quantum physics^1^,^2^,^3^,^4^,^5^,^6^. To date, diverse optomechanical structures^6^ have been developed on a variety of material platforms including silica^7^, silicon nitride^8^, silicon^9^, gallium arsenide^10^, aluminium nitride^11^, diamond^12^, phospho-silicate glass^13^, and gallium phosphide^14^. In general, cavity optomechanics relies critically on the underlying device material, requiring not only high optical transparency and large refractive index to support the high-quality and strong-confined optical modes, but also large acoustic velocity and low material damping to support the high-frequency and high-quality mechanical resonances.

Silicon carbide (SiC) is well known for its outstanding thermal, optical, mechanical and chemical properties^15^, with broad applications in high-power electronics, micromechanical sensors, biomedical devices, and astronomical telescopes^16^,^17^,^18^. In the past few years, significant efforts have been devoted to develop SiC-based micro/nanophotonic devices^19^,^20^,^21^,^22^,^23^,^24^,^25^,^26^,^27^,^28^,^29^,^30^, greatly attracted by its nonlinear optical properties^26^,^28^ and defect characteristics^31^,^32^. On the other hand, recent theoretical studies^33^,^34^,^35^ show that SiC exhibits intrinsic mechanical quality significantly superior than other materials, with a theoretical frequency-quality (f_m_ ⋅ Q_m_) product ~3 × 10^14^ at room temperature, due to its exceptionally low phonon-phonon scattering that dominates the intrinsic mechanical loss in the microwave frequency regime. The high intrinsic mechanical quality, together with the outstanding optical properties, makes SiC an excellent material platform for optomechanical applications. Unfortunately, the superior mechanical rigidity and chemical inertness of SiC impose significant challenge on fabricating micro-/nano-photonic devices with high optical and mechanical qualities, which seriously hinders the realization of optomechanical functionalities on the SiC platform.

In this letter, we demonstrate the first SiC optomechanical microresonators that exhibit significant optomechanical coupling with a coefficient up to |g_om_|/2*π* ≈ (61 ± 8) GHz/nm, which enables us to efficiently actuate and characterize the mesoscopic mechanical motions by optical means. By optimizing the device structure and the fabrication process, we are able to achieve high optical quality, large mechanical frequency, and high mechanical quality simultaneously in a single device. The whispering-gallery modes exhibit high optical qualities around ~3.8 × 10^4^. The radial-breathing mechanical modes show frequencies up to 1.69 GHz and mechanical qualities around 5500. The corresponding f_m_ ⋅ Q_m_ product is 9.47 × 10^12^, which is the highest value for the fundamental bulk acoustic mode in SiC demonstrated to date^36^,^37^,^38^,^39^,^40^,^41^,^42^,^43^,^44^,^45^,^46^,^47^, to the best of our knowledge.

The high performance of the demonstrated optomechanical microresonators shows that SiC devices are now ready for broad optomechanical applications. With the superior thermal and chemical properties of SiC material^15^, SiC optomechanical devices are particularly attractive for optomechanical sensing, such as displacement, force, mass, and inertial sensing, especially in harsh environments that are challenging for other device platforms. On the other hand, the SiC optomechanical microresonators, in combination with SiC’s significant optical nonlinearities^26^,^28^ and unique defect characteristics^31^,^32^, are of great promise for realizing hybrid micro/nanophotonic circuits for nano-optomechanics, integrated nonlinear photonics, and quantum photonics.

## Results

### Optomechanical device

**Figure 1:**
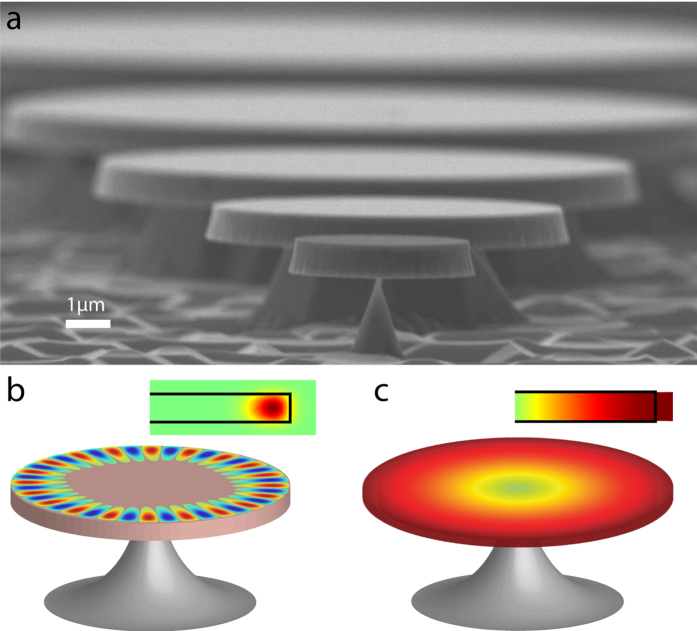
(**a**) Scanning electron microscope (SEM) image shows the fabricated 3C-SiC microdisks with different radii sitting on silicon pedestals. The pedestal of the smallest microdisk is critically controlled to optimize the mechanical quality of the radial-breathing mode. The smallest microdisk is darker due to the carbon deposition in the SEM process. (**b**,**c**) illustrate the mode profiles for a whispering-gallery optical mode and the fundamental radial-stretching mechanical mode, respectively, with the insets showing the cross-section view. Both mode profiles are simulated by finite-element methods.

The devices we employed are cubic-type (3C) silicon carbide (SiC) microresonators sitting on silicon pedestals. The device fabrication process is described in *Methods*. Figure 1(a) shows the fabricated devices of different radii with smooth sidewalls and fine-controlled undercuts. The fabrication process is optimized to produce smooth sidewalls, which are critical for minimizing the scattering loss of the optical modes. The device undercuts are optimized to reduce the clamping loss, which improves the mechanical qualities of the radial-breathing modes.

The microresonator exhibits whispering-gallery optical modes (Fig. 1b) that produce radiation pressure along the radial direction to actuate the fundamental radial-breathing mechanical modes (Fig. 1c), which in turn changes the cavity length and thus shifts the optical resonance frequency. The resulting dynamic backaction between the optical field and mechanical motion can be used to excite and probe the coherent mechanical motion, with efficiency dependent on the optomechanical coupling strength. For a microdisk optomechanical resonator with a radius of *r*, the optomechanical coupling coefficient scales as g_om_ ≈ −*ω*_o_/r, where *ω*_o_ represents the optical resonance frequency. The detailed simulations by the finite-element method (FEM) show that a SiC microdisk with a radius of 2 *μ*m and a thickness of 700 nm exhibits optomechanical coupling coefficients of |g_om_|/(2*π*) = 89 and 73 GHz/nm, respectively, for the fundamental and second-order transverse-electric-like (TE-like) modes, which correspond to a strong radiation pressure force of |ħg_om_| = 59 and 48 fN produced by each photon, respectively. The FEM simulation indicates that the fundamental radial-breathing mechanical mode of the device exhibits an effective motional mass of m_eff_ = 22 picograms. As a result, the vacuum optomechanical coupling rate, 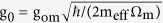, is as large as |g_0_|/(2*π*) = 42 kHz for the fundamental TE-like modes in the device.

### Optical Q characterization

**Figure 2:**
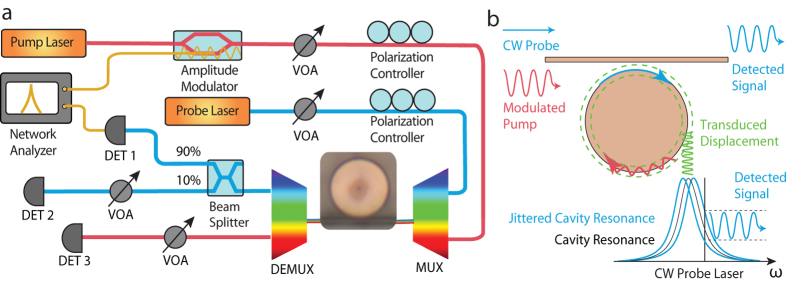
(**a**) The experimental setup for the optical pump-probe scheme. VOA, MUX, and DEMUX represent variable optical attenuator, multiplexer, and demultiplexer, respectively. (**b**) An illustration of the pump-probe scheme.

**Figure 3:**
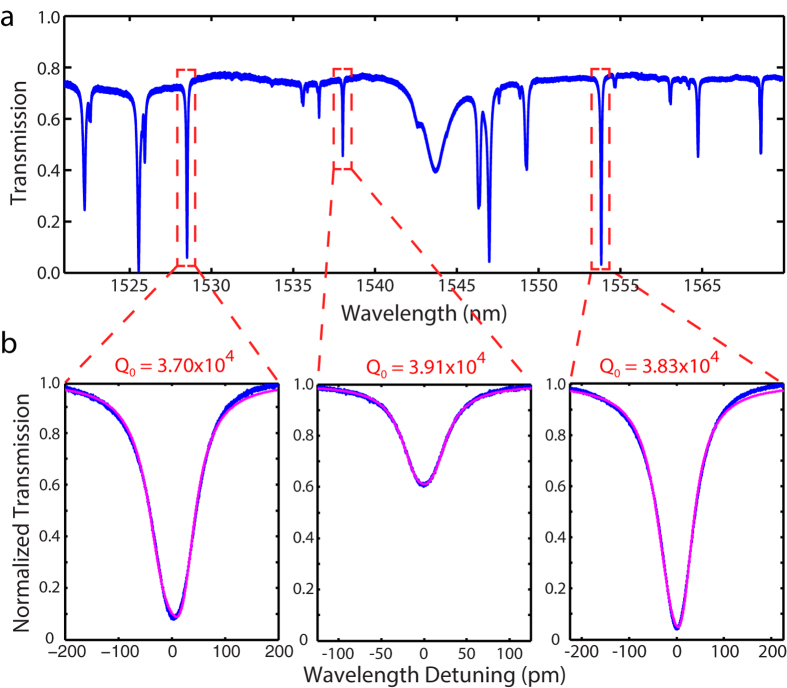
(**a**) Cavity transmission of a typical SiC optomechanical microresonator. (**b**) Three cavity modes have intrinsic optical qualities around 3.8 × 10^4^, with experimental data in blue and theoretical fitting in red.

The optical properties of devices are tested by a fiber-device coupling setup shown in Fig. 2. A tunable laser is launched into the devices by evanescent coupling through a tapered optical fiber. The cavity transmission is coupled out by the same tapered fiber and then recorded by fast detectors. The laser wavelength is calibrated by a Mach-Zehnder interferometer. A typical cavity transmission trace is shown in Fig. 3(a) with multiple high-Q optical modes. Three optical modes from different mode families all show optical qualities around 3.8 × 10^4^ (Fig. 3(b)). The coupling conditions of these modes can be easily tuned from under coupled, critical coupled to over coupled by tuning the fiber-device distance. For example, the cavity modes located around 1528 nm and 1553 nm are nearly critically coupled in this case.

### Optomechanical excitation and sensing

The high optical quality of the whispering gallery modes, combined with the strong optomechanical coupling, enables efficient excitation and probing of the mechanical motion. To do so, we launch an optical wave (the pump wave) into a cavity resonance, with power sinusoidally modulated at a frequency around the mechanical resonance frequency. The operation principle is illustrated in Fig. 2(b). A sinusoidal modulation of the optical power leads to a sinusoidally time varying radiation pressure that actuates the radial-breathing mechanical motion coherently via the strong optomechanical coupling. To probe such optomechanical excitation, we launch a weak continuous-wave optical wave (the probe wave) at a different cavity resonance. The coherent optomechanical excitation modulates the probe field inside the cavity via the optomechanical coupling, which is in turn transduced to the cavity output. Figure 2(a) shows schematically the experiment testing setup, with more detailed information given in the *Methods*. The devices are tested at room temperature in the atmospheric environment.

A detailed analysis of the optomechanical dynamics shows that the modulated probe power, *δP*_*s*_(Ω), at the modulation frequency Ω, detected at the cavity transmission is given by




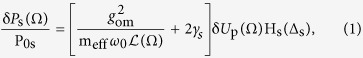




where *δ*U_p_(Ω) represents the modulated intra-cavity pump energy. H_s_(Δ_s_) is the cavity transduction function of the probe mode. The detailed expressions of *δ*U_p_(Ω) and H_s_(Δ_s_) can be found in ref. 26. Eq. (1) includes both optomechanical effect and optical Kerr effect. The first term describes the optomechanical response, with 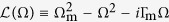 where Ω_m_ and Γ_m_ are the frequency and damping rate of the mechanical mode, respectively. The second term containing γ_s_ describes Kerr nonlinear response, with 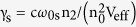 where n_0_ and n_2_ are the refractive index and Kerr nonlinear coefficient of SiC, respectively. ω_0s_ is the resonance frequency of the probe mode and V_eff_ represents the effective volume of the optical mode.

Our devices fall into the sideband unresolved regime, where the mechanical frequency is much smaller than the optical linewidth^6^. In this regime, Eq. (1) can be simplified to




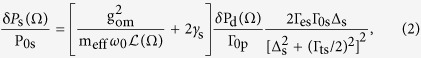




where *δ*P_d_(Ω) stands for the modulated pump power dropped inside the cavity. Γ_0p_ is the intrinsic photon decay rate of the pump mode. Γ_0s_ and Γ_ts_ represent intrinsic and total photon decay rate of the probe mode, respectively. Γ_es_ represents its external coupling rate. Δ_s_ = *ω*_s_ − *ω*_0s_ is the laser-cavity detuning of the probe wave.

In the experiments, the optical mode is typically near critical-coupling conditions, Γ_0s_ = Γ_es_, and the laser detuning for the probe mode is set around the half of total cavity linewidth Δ_s_ ~ Γ_ts_/2. As a result, Eq. 2 reduces considerably to




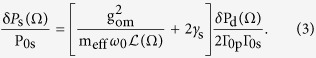




Equation (3) clearly shows the linear dependence of the transduced probe signal on the optical qualities of the pump and probe modes. Moveover, it depends quadratically on the optomechanical coupling coefficient g_om_ since the optomechanical effect not only drives the mechanical mode by the modulated pump beam, but also transduces the mechanical motion to the probe beam. Consequently, significant optomechanical coupling and high optical quality in the devices would lead to efficient optomechanical excitation and transduction by the pump and probe waves.

**Figure 4:**
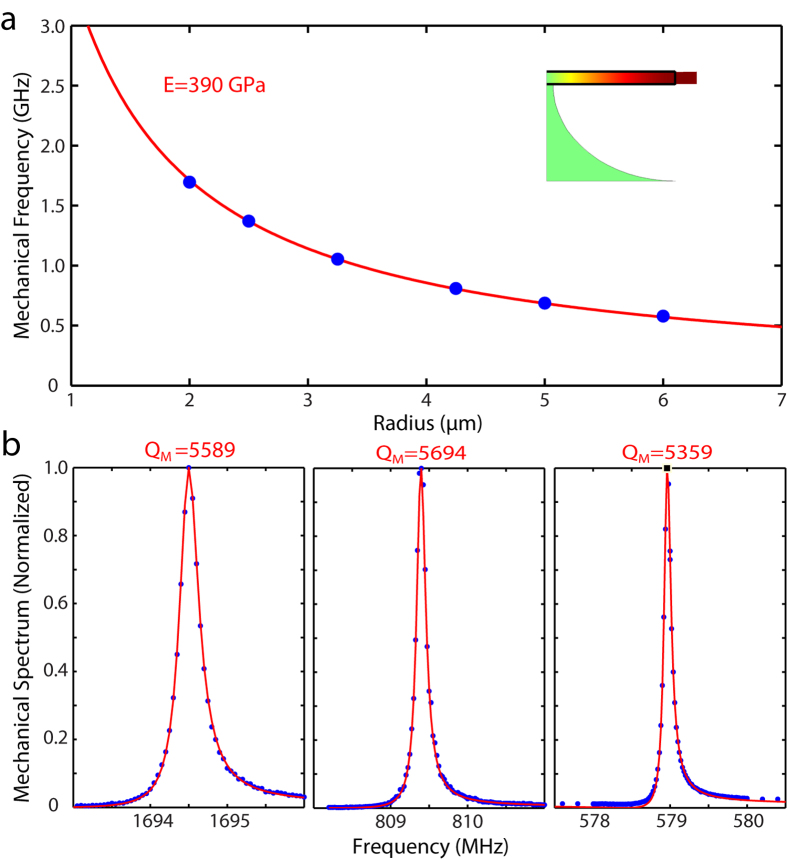
(**a**) Mechanical frequencies of the fundamental mechanical radial-stretching modes are inversely proportional to the radii of the microdisks. Experimental dots are in blue and the fitting curve is in red. Inset represents the displacement of a typical fundamental mechanical radial-stretching mode, with the geometrical edges outlined in black. (**b**) Normalized mechanical transduction spectra of the silicon carbide microdisks with radii being 2, 4.25, and 6 *μ*m, shown from left to right. Experimental dots are in blue and fitting curves are in red. The data are fitted by Eq. 2. The silicon carbide microdisks maintain high mechanical Q factors around 5,500 for all the devices.

Equations (1)–(3), 5, 6 show that, by scanning the modulation frequency, we can obtain the mechanical response of the radial-breathing mode. Figure 4(b) shows three examples of devices with different radii of 2, 4.25, and 6 *μ*m, respectively. The radial-breathing mechanical modes exhibit distinctive mechanical frequencies in these devices but all with a mechanical Q above 5000. The slight spectral asymmetry on the mechanical spectra is primarily due to the Fano-type interference between the narrow-band mechanical response and the broadband background of optical Kerr nonlinear response (see Eq. (2)). A comparison of the recorded optomechanical spectra with the theory infers an optomechanical coupling coefficient of |g_om_|/(2*π*) = (61 ± 8) GHz/nm for the 2 *μ*m device. This is smaller than the FEM simulated value (89 GHz/nm), which accounts for the radiation pressure of the shifting dielectric boundary. The discrepancy is likely from the electrostrictive contribution in the dielectric material^48^. We also characterize the devices with different radii to map out the dependence of mechanical frequency. As shown in Fig. 4(a), the mechanical frequency of the radial-breathing mode scales inversely with the device radius. Comparing the experimental data (blue dots) with the theoretical prediction (red curve), we infer the Young’s modulus to be 390 GPa, which is consistent with previous measurements of 3C-SiC epitaxial films on silicon substrates^49^.

**Figure 5:**
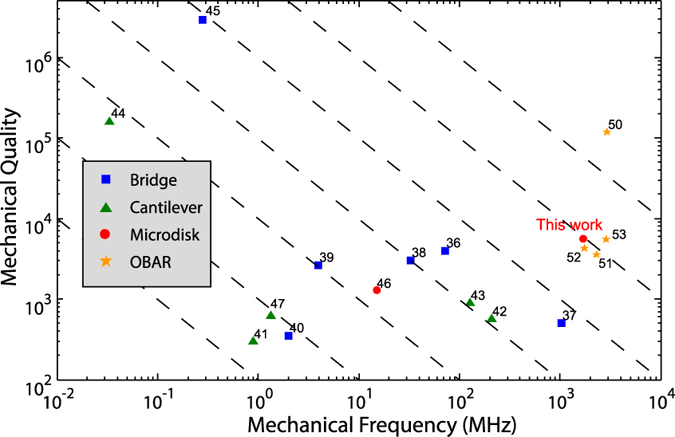
The frequency-quality products of the SiC mechanical resonantors. Blue squares, green triangles, red circles, and yellow stars represent bridges, cantilevers, microdisks, and overtone bulk acoustic resonators (OBARs), respectively. The dashed black lines show the equal f_m_ ⋅ Q_m_ product lines from 10^14^ Hz (top right) to 10^8^ Hz (bottom left).

One critical figure of merit for mechanical resonators is the f_m_ ⋅ Q_m_ product, which quantifies the degree of decoupling of mechanical motion from the environmental thermal reservoir^6^. Figure 5 summarizes the f_m_ ⋅ Q_m_ product reported to date for SiC micro/nanomechanical resonators^36^,^37^,^38^,^39^,^40^,^41^,^42^,^43^,^44^,^45^,^46^,^47^,^50^,^51^,^52^,^53^. In general, bridge- and cantilever-type SiC micro/nanomechanical resonators exhibit low f_m_ ⋅ Q_m_ products, with a mechanical damping dominated by the mechanical clamping loss. To mitigate the clamping loss, high-order overtone-bulk-acoustic-resonator (OBAR) modes are employed to store mechanical energy over many mechanical wavelengths^50^,^51^,^52^,^53^, which, however, requires a large device size significantly greater than the mechanical wavelength that seriously limits the device miniaturization and integration.

In contrast, our optomechanical resonators operate in the fundamental radial-breathing acoustic mode, with a small device size comparable to the mechanical wavelength. For example, the device with a radius of 2 *μ*m exhibits a frequency of 1.69 GHz and a mechanical Q of 5589 (Fig. 4(b)), which corresponds to a f_m_ ⋅ Q_m_ product of 9.47 × 10^12^ Hz. This product is among the largest values reported up to date of SiC devices^36^,^37^,^38^,^39^,^40^,^41^,^42^,^43^,^44^,^45^,^46^,^47^,^50^,^51^,^52^,^53^, as shown in Fig. 5. In fact, our device has the largest f_m_ ⋅ Q_m_ product among whispering-gallery-type optomechanical microresonators made from various materials^7^,^10^,^11^,^13^,^14^,^54^,^55^, as shown in Table 1. This value is still about an order of magnitude lower than the theoretical f_m_ ⋅ Q_m_ product^33^,^34^,^35^, implying that the current limitation is not on intrinsic mechanical loss of SiC material, but on practical factors such as device etching, pillar clamping, and air damping. We thus expect improvement of the f_m_ ⋅ Q_m_ product in the future after further optimization of the device structure and fabrication process. Table 1 also shows that current SiC devices have lower optical qualities than the state-of-the-art optomechanical devices in other materials. We are currently optimizing the fabrication process to improve the optical quality of SiC for practical optomechanical applications.

## Discussions

We have demonstrated the first SiC optomechanical resonators in 3C-SiC microdisks that exhibit strong optomechanical coupling and excellent mechanical qualities, with a f_m_ ⋅ Q_m_ product as high as 9.47 × 10^12^ Hz. The high performance of the demonstrated devices infers that the SiC optomechanical devices are of great potential for metrology and sensing applications, particularly in detecting displacement, force, mass, and acceleration/rotation with high sensitivity. In combination with SiC’s superior thermal property, chemical inertness, hand high breakdown voltage, SiC optomechanical devices are of great promise for applications in various harsh environments, such as those with high temperature, reactive chemicals, biological fluid, or high electric field^15^,^16^,^42^,^56^,^57^,^58^, that are challenging for other device platforms.

On the other hand, the SiC optomechanical microresonators exhibit a mechanical frequency scalable by the device radius. In particular, the SiC microdisk with a radius of 2.5 *μ*m exhibits a mechanical frequency of 1.33 GHz (see Fig. 4), which matches the zero-field splitting of spin ground states of the point defects in 3C-SiC^31^,^32^. Therefore, the high-Q collective mechanical mode is potentially able to coherently interact with the ground states of the defect spin via stress-induced coupling. This mechanism, in combination with the photon-spin coupling in SiC^24^,^25^ and photon-photon interaction via SiC’s significant χ^(2)^ and χ^(3)^ nonlinearities^26^,^28^, is of great potential to form a hybrid micro-/nano-photonic circuit that mutually couples photon, defect spin, and acoustic phonon for nonlinear optical, quantum optical, and optomechanical functionalities.

## Methods

### Device fabrication

The device structure we employed is cubic-polytype silicon carbide (3C-SiC) microdisks sitting on silicon pedestals. A high-definition electron-beam resist (ZEP520A) is used to pattern Chromium (Cr) mask with chlorine-based plasma by reactive-ion etching (RIE). The Cr mask is later used as a hard mask to etch SiC with fluorine-based plasma by inductively coupled-plasma RIE. The residue of Cr is then released by CR-14, a Cr etchant, and the silicon substrate is undercut by potassium hydroxide. The device is annealed afterwards at 1100 °C for 2 hours. Figure 1 shows the fabricated devices of different radii with smooth sidewalls and fine-controlled undercuts. More fabrication details can be found in ref. 25.

### Pump-probe setup

The experimental setup is shown in detail in Fig. 2(a). An intensive laser wave is sinusoidally modulated in amplitudes by a lithium niobate modulator. The frequency of modulation is scanned by a network analyzer. The pump laser is attenuated by a variable optical attenuator (VOA) to ~80 *μ*W. The probe laser is kept 10 dB smaller than the pump beam by another VOA. The thermal effect is negligible for the operating powers in the devices. The polarization controllers are used to change the polarizations of the laser beams to the employed cavity modes. A coarse-wavelength-division-multiplexing (CWDM) multiplexer is used to combine the pump and probe beams and launch them into the cavity. The modulated pump beam drives the mechanical mode, with the mechanical displacement transduced to the jittering of the cavity resonance frequencies. The pump and probe beam are then separated by the CWDM demultiplexer. Detector 1, with 90% transmission of probe beam, is collected by the network analyzer. The network analyzer scans the modulation frequencies and detects the signal at the same frequencies simultaneously. Detectors 2 and 3 are used for locking laser cavities to probe and pump modes, respectively. The optical modes we employed in the experiments are high order modes, which can be easily critically coupled by the current tapered fiber. The optomechanical coupling can be improved by accessing the fundamental modes through thinner tapered fiber or waveguide coupling.

## Additional Information

**How to cite this article**: Lu, X. *et al.* High-frequency and high-quality silicon carbide optomechanical microresonators. *Sci. Rep.* **5**, 17005; doi: 10.1038/srep17005 (2015).

## Figures and Tables

**Table 1:** Typical physical parameters for whispering-gallery-type optomechanical microresonators.

Material	f_m_ · Q_m_(GHz)	f_m_(GHz)	Q_m_	m_eff_(pg)	Q_o_	|g_om_|/(2*π*) (GHz/nm)	x_0_ (fm)	|g_0_|/(2*π*) (kHz)
Silicon Carbide^†^	9470	1.69	5589	22	3.9 × 10^4^	61	0.47^c^	29^c^
Silica^7^	15.4^c^	0.0044	3500	33	>1 × 10^7^	5.57^r^	7.607^c^	41.87^c^
Silicon^54^	4320	1.294	3300	5.7	3.5 × 10^5^	115	0.947^c^	1087^c^
Silicon Nitride^55^	6250	0.625	10000	677^s^	~1 × 10^6^	177^c^	0.457^c^	7.8
Aluminum Nitride^11^	25707^c^	1.04	2473	420	1.25 × 10^5^	5.37^r^	0.147^c^	0.77^c^
Gallium Phosphide^14^	3127^c^	0.488	~640	40	2.6 × 10^5^	48	0.657^c^	31
Gallium Arsenide^10^	40.3	0.336	120	22.1	~1 × 10^5^	95	1.06^c^	101
Phosphosilicate Glass^13^	22.3^c^	0.019	1200	11800	1.5 × 10^6^	3.87^r^	0.207^c^	0.7

^†^This work, ^s^simulated value, ^r^estimated by g_om_ ≈ −*ω*_o_/r, ^c^calculated value.

Other values without superscripts are directly from corresponding references.
